# Completion of the Entire Hepatitis C Virus Life Cycle in Vero Cells Derived from Monkey Kidney

**DOI:** 10.1128/mBio.00273-16

**Published:** 2016-06-14

**Authors:** Asako Murayama, Nao Sugiyama, Takaji Wakita, Takanobu Kato

**Affiliations:** Department of Virology II, National Institute of Infectious Diseases, Tokyo, Japan

## Abstract

A hepatitis C virus (HCV) cell culture system incorporating the JFH-1 strain and the human hepatoma cell line HuH-7 enabled the production of infectious HCV particles. Several host factors were identified as essential for HCV replication. Supplementation of these factors in nonhepatic human cell lines enabled HCV replication and particle production. Vero cells established from monkey kidney are commonly used for the production of vaccines against a variety of viruses. In this study, we aimed to establish a novel Vero cell line to reconstruct the HCV life cycle. Unmodified Vero cells did not allow HCV infection or replication. The expression of microRNA 122 (miR-122), an essential factor for HCV replication, is notably low in Vero cells. Therefore, we supplemented Vero cells with miR-122 and found that HCV replication was enhanced. However, Vero cells that expressed miR-122 still did not allow HCV infection. We supplemented HCV receptor molecules and found that scavenger receptor class B type I (SRBI) was essential for HCV infection in Vero cells. The supplementation of apolipoprotein E (ApoE), a host factor important for virus production, enabled the production of infectious virus in Vero cells. Finally, we created a Vero cell line that expressed the essential factors miR-122, SRBI, and ApoE; the entire HCV life cycle, including infection, replication, and infectious virus production, was completed in these cells. In conclusion, we demonstrated that miR-122, SRBI, and ApoE were necessary and sufficient for the completion of the entire HCV life cycle in nonhuman, nonhepatic Vero cells.

## IMPORTANCE

HCV is a major cause of chronic liver diseases worldwide, and an effective prophylactic HCV vaccine is needed. For safety reasons, the current HCV cell culture system using HuH-7 cells, which was established from a hepatocellular carcinoma, is not suitable for the production of a vaccine against HCV. A robust HCV production system using non-cancer-derived cells is indispensable for this purpose. In this study, we wanted to establish a novel HCV cell culture system using Vero cells, which are widely used in the production of vaccines against different viruses. We identified the minimum essential host factors for the completion of the entire HCV life cycle in Vero cells to develop a novel HCV cell culture system. A cell culture system that uses Vero cells will be useful not only for HCV vaccine production but also for the further elucidation of the mechanisms of various HCV-host interactions.

## INTRODUCTION

Hepatitis C virus (HCV) is a major cause of chronic liver diseases such as chronic hepatitis, liver cirrhosis, and hepatocellular carcinoma (1–3). The majority of HCV-infected patients, even adults, fail to clear this virus. Approximately 200 million people worldwide are currently infected with HCV and are at continued risk for the development of chronic liver diseases (4). An interferon (IFN)-based therapy has been used both to control disease progression and to eradicate HCV (5, 6). Novel direct-acting antiviral agents have been developed, and the number of patients in whom HCV has been eradicated has thus increased (7–9). However, these newly developed drugs are expensive and have associated side effects, and drug resistance is emerging. Vaccination is the most cost-efficient strategy to reduce the burden of viral infection all over the world. At present, no practical vaccine against HCV for prophylactic or therapeutic use exists.

The lack of a cell culture system capable of producing virus particles has hampered progress of HCV research. In 2005, a robust HCV cell culture system was developed using the HCV JFH-1 strain and HuH-7 cells, which were established from a hepatocellular carcinoma (10, 11). The JFH-1 strain was cloned from a fulminant hepatitis patient and could replicate autonomously and produce HCV particles *in vitro* (10, 12, 13), thereby facilitating investigation of the entire life cycle of HCV. Many host factors associated with the HCV life cycle have been identified, and some of them were considered the essential factors for HCV infection, replication, and virus production in hepatocytes (14–17). By supplementing these factors in nonhepatic cells, HCV production became possible in cells other than Huh-7 cells. For example, HEK293T cells are human kidney-derived cells, and the ectopic expression of microRNA 122 (miR-122) and Claudin-1 (CLDN1) enabled HCV replication after HCV infection of HEK293T cells (16). FU97 cells, derived from stomach cancer, and originally expressing essential host factors for HCV life cycle at levels comparable to those of HuH-7 cells, support HCV replication after HCV infection (18). These observations indicate the possibility that any cell line could allow HCV replication if it expressed the appropriate host factors.

Cell culture-generated HCV (HCVcc) has been reported to be a promising candidate for a prophylactic vaccine against HCV (19). For the purpose of HCVcc vaccine development, a cell culture system using non-tumor-derived cells is indispensable in reducing the risk of contamination of some hidden oncogenic agents that might be transferred to vaccine recipients. Because the current HCV cell culture system was developed using a human hepatoma-derived cell line, virus particles produced from these cells are not suitable for vaccine development. Vero cells were established from the monkey kidney and are commonly used for the production of vaccines against a variety of viruses (20). In this study, we identified the host factors in Vero cells essential for the completion of the entire HCV life cycle and developed a novel HCV cell culture system.

## RESULTS

### HCV replication in Vero cells.

**FIG 1:**
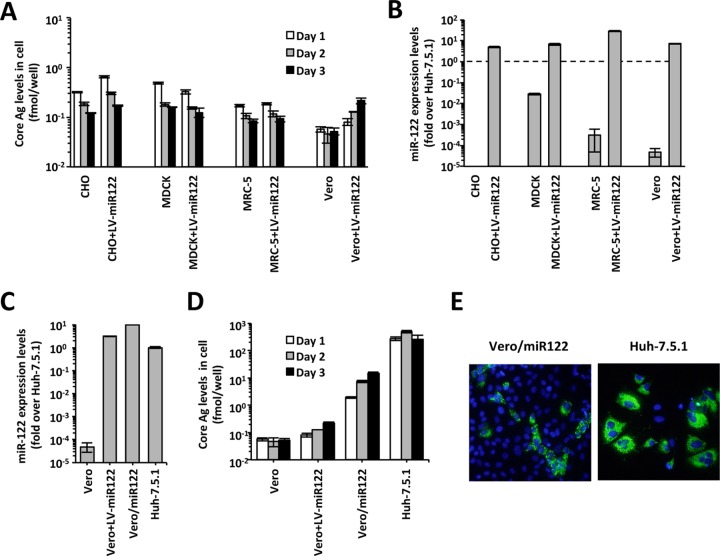
HCV replication in Vero cells expressing miR-122. (A) HCV replication after HCV RNA transfection. HCV RNA was electroporated into CHO cells, MDCK cells, MRC-5 cells, and Vero cells. The HCV core protein levels in the cell lysates were measured. (B) Comparisons of the miR-122 expression levels in CHO cells, MDCK cells, MRC-5 cells, and Vero cells. Quantitative PCR was performed using gene-specific primers and probe sets for U6 and miR-122. The data are expressed as the fold difference in expression compared to the expression level in Huh-7.5.1 cells. The level of expression of miR-122 in Huh-7.5.1 cells is indicated by the dashed line. (C) Comparison of the miR-122 expression levels in Vero cells and in Huh-7.5.1 cells. Data are expressed as the fold difference in expression compared to the expression level in Huh-7.5.1 cells. (D) HCV replication in Vero cells expressing miR-122. The HCV core protein levels in the cell lysates were measured. (E) HCV-positive cells after HCV RNA transfection. HCV-positive cells were visualized with anti-NS5A antibody (green), and nuclei were visualized with 4′,6′-diamidino-2-phenylindole (DAPI) (blue).

To establish a novel HCV cell culture system using non-cancer-derived cells, we tested the susceptibility to HCV infection and replication in several vaccine producer cell lines: CHO cells (derived from Chinese hamster ovary), MDCK cells (derived from canine kidney), MRC-5 cells (derived from healthy human lung tissue), and Vero cells (derived from monkey kidney). However, no HCV-positive cells were detected in these cells after HCVcc infection (data not shown). After HCV RNA transfection into these cells, a time-dependent increase in the HCV core antigen (Ag) was not observed, indicating that these cells did not support HCV replication (Fig. 1A). From the above results, we concluded that unmodified CHO cells, MDCK cells, MRC-5 cells, and Vero cells are not susceptible for HCV infection and replication.

The liver-specific miR-122 is an important host factor for HCV replication. Because the miR-122 expression level in these cells was quite low compared to the levels in Huh-7.5.1 cells (Fig. 1B), we introduced miR-122 into these cells via lentiviral transduction. The miR-122 expression level in these cell lines became higher than that in Huh-7.5.1 cells after lentiviral transduction (Fig. 1B). Then, we tested the susceptibility for HCV replication in these cells in which miR-122 had been introduced. A slight, time-dependent increase in the HCV core Ag was observed in miR-122-transduced Vero cells, indicating that Vero cells expressing miR-122 supported HCV replication at a low level after HCV RNA transfection (Vero+LV-miR122 [LV stands for lentivirus] in Fig. 1A).

To establish a Vero cell line that supported more efficient HCV replication, we performed single-cell cloning of miR-122-transduced bulk Vero cells (Vero+LV-miR122). Because the cell clones obtained expressed miR-122 at different levels, we selected the Vero cell clone that had the highest miR-122 expression level and designated it Vero/miR122. The expression level of miR-122 in Vero/miR122 cells was approximately 10-fold higher than that in Huh-7.5.1 cells (Fig. 1C). After HCV RNA transfection, the HCV core Ag level in Vero/miR122 cells increased in a time-dependent manner and was 70 times higher than that in Vero+LV-miR122 cells but still 17.7-fold lower than that in Huh-7.5.1 cells at 3 days after transfection (Fig. 1D). HCV NS5A-positive Vero cells were detected via immunostaining at 3 days after HCV RNA transfection (Fig. 1E), indicating that Vero/miR122 cells supported efficient HCV replication after HCV RNA transfection.

### HCV infection of Vero cells.

**FIG 2:**
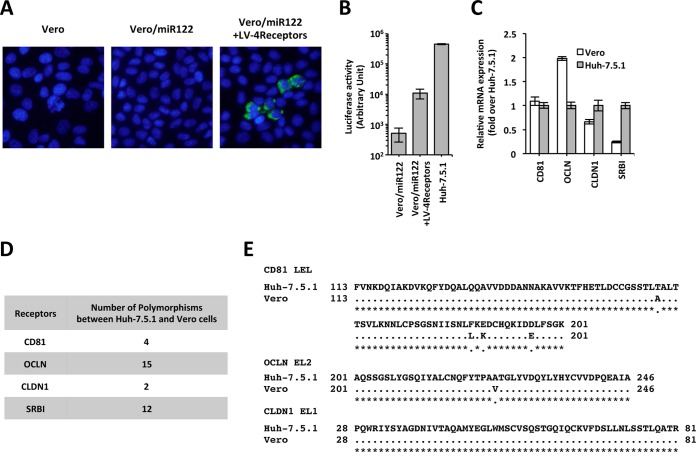
HCV infection of Vero cells. (A) HCVcc infection into Vero cells expressing miR-122 and four HCV receptors. Vero cells, Vero/miR122 cells, and Vero/miR122+LV-4Receptors cells were infected with HCVcc, HCV-positive cells were visualized with anti-NS5A antibody (green), and nuclei were visualized with DAPI (blue). (B) HCVpp infection in Vero cells expressing miR-122 and four HCV receptors. The luciferase activity in the cell lysate was quantified. (C) Gene expression levels of HCV receptors in Vero cells. The results are expressed as the fold difference in expression compared to the level in Huh-7.5.1 cells. (D) Number of polymorphisms between Huh-7.5.1 cells and Vero cells. (E) Sequence alignment of CD81 LEL (large extracellular loop), OCLN EL2 (extracellular loop 2), and CLDN1 EL1.

Though Vero/miR122 cells supported HCV replication, HCV-positive cells were not observed after HCVcc infection (Fig. 2A). Then, we introduced four human HCV receptors, CD81, occludin (OCLN), CLDN1, and scavenger receptor class B type I (SRBI), into Vero/miR122 cells via lentiviral transduction. Four HCV receptor-transduced bulk Vero cells, designated Vero/miR122+LV-4Receptors, became susceptible to infection with HCVcc (Fig. 2A) and HCV pseudotype virus (HCVpp) (Fig. 2B). To identify the molecule responsible for this susceptibility, we investigated the expression level and polymorphism of these receptors in Vero cells. To evaluate the expression level of each receptor in Vero cells, we measured the mRNA expression level. The expression level of SRBI in Vero cells was markedly lower than that in Huh-7.5.1 cells (24% of Huh-7.5.1 cells) (Fig. 2C). The expression levels of CD81 and OCLN in Vero cells were comparable or higher than that in Huh-7.5.1 cells, and the expression level of CLDN1 was slightly lower than that in Huh-7.5.1 cells (66% of Huh-7.5.1 cells) (Fig. 2C). To evaluate the amino acid polymorphisms, we sequenced the mRNAs of these HCV receptors in Huh-7.5.1 and Vero cells by reverse transcription-PCR (RT-PCR). We found four polymorphisms in the CD81 open reading frame (ORF) (Fig. 2D), all of which were located in the large extracellular loop (LEL) region (Fig. 2E), which has been reported to be important for the association with HCV E2 protein (21). Fifteen polymorphisms were found in the OCLN ORF (Fig. 2D), and one was located in the extracellular loop 2 (EL2) region (Fig. 2E), which has been reported to be important for the association with HCV E2 protein (22). Two polymorphisms were found in the CLDN1 ORF (Fig. 2D); however, none was located in the EL1 region (Fig. 2E), which has been reported to be important for HCVpp entry (23). Twelve polymorphisms were found in the SRBI ORF (Fig. 2D), located throughout the ORF. These data indicated that the lower expression of SRBI and/or polymorphisms in each HCV receptor molecule were considered to be responsible for the nonsusceptibility of Vero cells to HCV infection.

To confirm whether the HCV receptor molecules in Vero cells were functional upon HCV infection, we cloned CD81, OCLN, CLDN1 and SRBI from Vero and Huh-7.5.1 cells and assessed their contribution to HCV infection via transduction into cell lines that lacked the expression of these molecules. To analyze the function of CD81, we used Huh7-25 cells that lacked CD81 expression (Huh7-25 [see Fig. S1A in the supplemental material]). We introduced Vero- or human-derived CD81 (vCD81 or hCD81, respectively) into the Huh7-25 cells via lentiviral transduction. To analyze the function of OCLN, we used 786-O cells that lacked OCLN mRNA expression (786-O [see Fig. S1A in the supplemental material]). The expression of miR-122 was also lacking in 786-O cells, and the ectopic expression of miR-122 (786-O/miR122) did not allow HCV replication after HCV RNA transfection (Fig. S1B and S1C). We introduced Vero- or Huh-7.5.1-derived OCLN (vOCLN or hOCLN, respectively) into 786-O/miR122 cells and used these cells only for the HCVpp infection assay. To analyze the function of CLDN1, we used HEK293 cells that expressed CLDN1 at a low level (Fig. S1A, HEK293). HEK293 cells also do not express miR-122. The ectopic expression of miR-122 enabled HCV replication after HCV RNA transfection (HEK293/miR122 [Fig. S1B and S1D]). We introduced Vero- or Huh-7.5.1-derived CLDN1 (vCLDN1 or hCLDN1, respectively) into HEK293/miR122 cells via lentiviral transduction. We used Vero cells to analyze the function of SRBI because the expression level of SRBI was quite low (Fig. 2C). We introduced Vero- or Huh-7.5.1-derived SRBI (vSRBI or hSRBI, respectively) into Vero/miR122 cells via lentiviral transduction.

**FIG 3:**
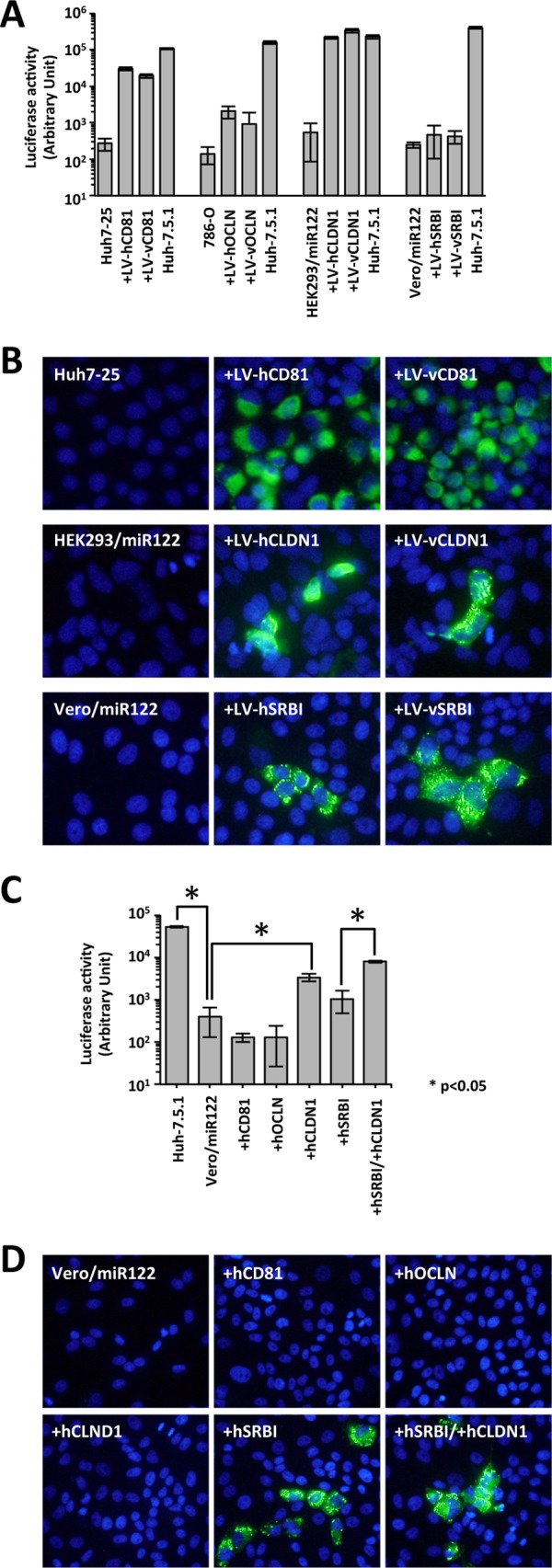
HCV infection of human- and Vero-derived HCV receptor-expressing cells. (A) HCVpp infection of Huh7-25 cells expressing human or viral CD81 (hCD81 or vCD81, respectively), 786-O cells expressing hOCLN or vOCLN, HEK293 cells expressing hCLDN1 or vCLDN1, and Vero cells expressing hSRBI or vSRBI. (B) HCVcc infection of Huh7-25 cells expressing hCD81 or vCD81, HEK293 cells expressing hCLDN1 or vCLDN1, and Vero cells expressing hSRBI or vSRBI. HCV-positive cells were visualized with anti-NS5A antibody (green), and the nuclei were visualized with DAPI (blue). (C) HCVpp infection of Vero cells expressing hCD81, hOCLN, hCLDN1, or hSRBI. (D) HCVcc infection of Vero cells expressing hCD81, hOCLN, hCLDN1, or hSRBI. HCV-positive cells were visualized with anti-NS5A antibody (green), and nuclei were visualized with DAPI (blue).

In the infection studies, inconsistent data were recorded for HCVpp and HCVcc. In the experiments with HCVpp, HCVpp infection was completely recovered by both hCLDN1 and vCLDN1 expression in HEK293/miR122 cells and partially recovered by both hCD81 and vCD81 expression in Huh7-25 cells (Fig. 3A). Both hOCLN and vOCLN expression slightly enhanced HCVpp infection in 786-O cells. The expression of either hSRBI or vSRBI did not enhance the susceptibility to HCVpp infection in Vero/miR122 cells. In the experiments with HCVcc, the HCV-infected foci were detected by both hCD81 and vCD81 expression in Huh7-25 cells (Fig. 3B, top panels). Similar results were also observed in both hCLDN1- and vCLDN1-transduced HEK293/miR122 cells (Fig. 3B, middle panels) and in both hSRBI- and vSRBI-transduced Vero/miR122 cells (Fig. 3B, bottom panels). The number of the HCV-infected foci of Fig. 3B was presented in Fig. S2A in the supplemental material. These results indicated that the HCV receptor molecules originally expressed in Vero cells could function as HCV receptors. We judged that the ectopic expression of both hSRBI and vSRBI in HCVcc was sufficient for susceptibility to HCVcc infection in Vero cells. Because the CLDN1 expression level was slightly lower in the Vero cells than in the Huh-7.5.1 cells (Fig. 2C), we also investigated the effect of hCLDN1 expression in Vero/miR122 cells (Vero/miR122+LV-hCLDN1) on HCVpp and HCVcc infection. Although hCLDN1 expression significantly enhanced HCVpp infection (Fig. 3C), it did not enable HCVcc infection in Vero/miR122 cells (Fig. 3D). Furthermore, additional hCLDN expression in Vero/miR122+LV-hSRBI cells enhanced HCVpp infection but did not increase the number of HCV-positive cells after HCVcc infection (Fig. 3C and D and Fig. S2B). Overexpression of hCD81 or hOCLN in Vero/miR122 did not enhance HCVpp or HCVcc infection (Fig. 3C and D and Fig. S2B). Taken together, these results indicated that the HCV receptors originally expressed in Vero cells could function as HCV receptors when they were expressed at a sufficiently high level, and the nonsusceptibility of Vero cells to HCVcc was due to the low expression of SRBI. These results also indicated that SRBI expression was necessary and sufficient for susceptibility to HCV infection in Vero cells.

### Infectious virus production in Vero cells.

**FIG 4:**
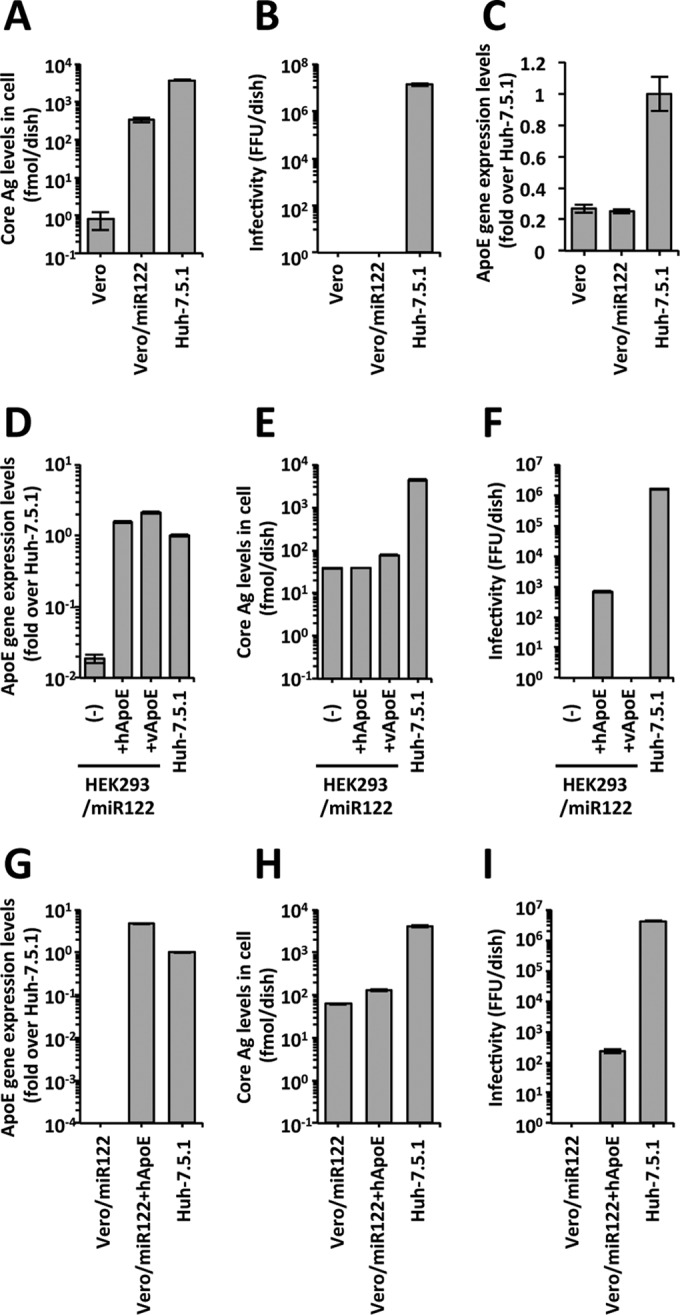
Infectious virus production of HCV from Vero cells expressing ApoE. (A, E, and H) HCV core antigen levels in cells that had been transfected with HCV RNA. The cells were harvested at day 3 (day 4 for HEK293 cells). (B, F, and I) Infectious titers in the culture media from HCV RNA-transfected cells. The culture medium collected on day 3 (day 4 for HEK293 cells) was concentrated, serially diluted, and added to naive Huh-7.5.1 cells. The inoculated cells were fixed, and the HCV-infected foci were visualized via staining with anti-NS5A antibody. (C, D, and G) Relative gene expression level of ApoE. The probe and primer sets that detected both human and monkey ApoE (C and D) and that detected only human ApoE (G) were used. Data are expressed as the fold difference in expression compared to the level in Huh-7.5.1 cells. FFU, focus-forming units.

Although Vero/miR122 cells supported HCV replication after HCV RNA transfection (Fig. 4A), no infectivity was detected in the cell culture medium (Fig. 4B). The mRNA expression level of apolipoprotein E (ApoE) was low in parental Vero and Vero/miR122 cells compared with Huh-7.5.1 cells (Fig. 4C). We determined the sequence of ApoE originally expressed in Vero cells and found 16 amino acid polymorphisms compared to Huh-7.5.1 cells. To assess the function of ApoE originally expressed in Vero cells, we cloned Vero- and human-derived ApoE (vApoE and hApoE, respectively) and compared the effects of these molecules on infectious virus production by introducing them into HEK293/miR122 cells because ApoE expression in these cells was negligible. The expression levels of ApoE in HEK293/miR122+hApoE and HEK293/miR122+vApoE cells were comparable to that in Huh-7.5.1 cells (Fig. 4D). After HCV RNA transfection, intracellular HCV core Ag levels in HEK293/miR122 and HEK293/miR122+hApoE cells were similar and slightly lower than in HEK293/miR122+vApoE cells (Fig. 4E). However, infectivity could be detected only in the medium of HEK293/miR122+hApoE cells (Fig. 4F). These results indicated that ApoE originally expressed in Vero cells did not allow infectious virus production of HCV.

Then, we established a Vero cell clone that expressed hApoE in addition to miR-122 (Vero/miR122+hApoE). To measure the mRNA expression of hApoE in Vero cells, we used a gene expression assay that detected human ApoE but not monkey ApoE. The expression level of hApoE in Vero/miR122+hApoE cells was higher than in Huh-7.5.1 cells (Fig. 4G). After HCV RNA transfection, the intracellular HCV core Ag level in Vero/miR122+hApoE cells was slightly higher than that in Vero/miR122 cells (Fig. 4H). Infectivity was detected in the medium of Vero/miR122+hApoE cells, although the titer was lower (by approximately 4 log units) than in Huh-7.5.1 cells (Fig. 4I). These results indicated that human ApoE expression allowed infectious virus production of HCV in Vero cells.

### Completion of the entire HCV life cycle in Vero cells.

**FIG 5:**
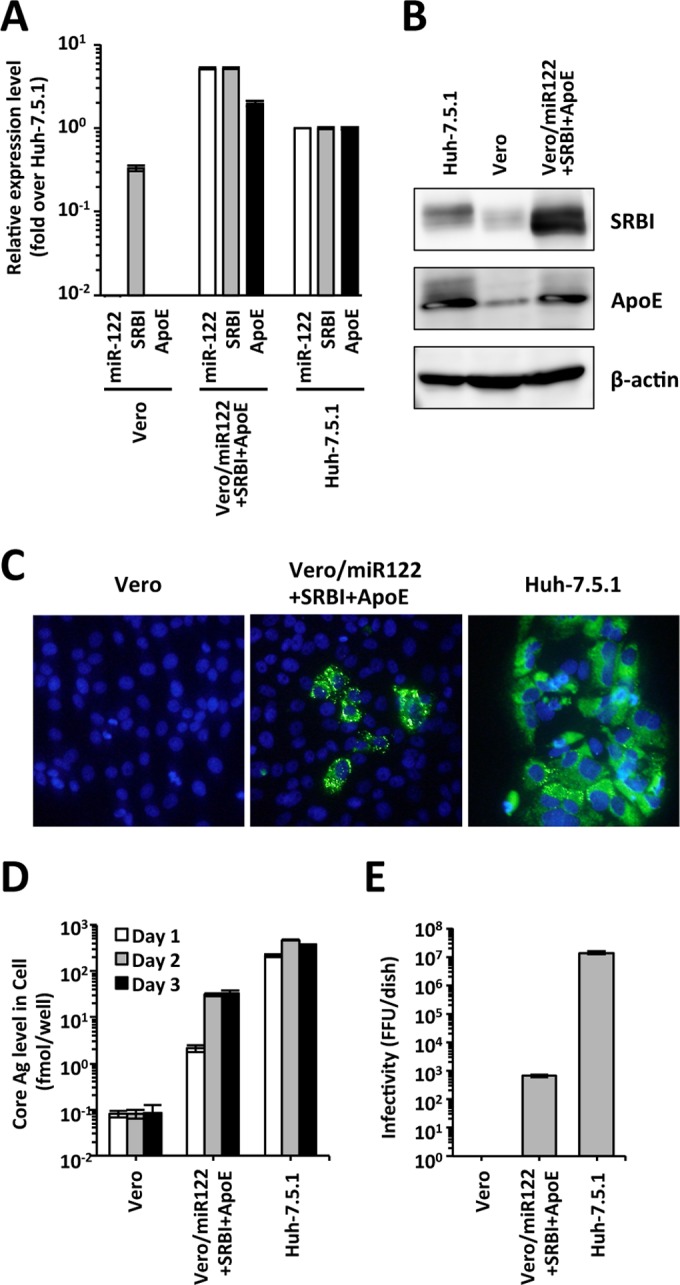
Coexpression of miR-122, SRBI, and ApoE enabled HCV infection, replication, and virus production in Vero cells. (A) Relative gene expression levels of miR-122, SRBI, and ApoE. The primer and probe set for miR-122 and SRBI detected the human and monkey orthologues, and the primer and probe set for ApoE detected only human ApoE. The results are expressed as the fold difference in expression compared to the level in Huh-7.5.1 cells. (B) SRBI and ApoE expression levels in Vero/miR122+SRBI+ApoE cells. Huh-7.5.1, Vero, and Vero/miR122+SRBI+ApoE cells were lysed, and protein expression was confirmed by Western blotting. (C) HCVcc infection of Vero, Vero/miR122+SRBI+ApoE, and Huh-7.5.1 cells. The HCV-positive cells were visualized with anti-NS5A antibody (green), and nuclei were visualized with DAPI (blue). (D) HCV RNA was electroporated into the cells, and the HCV core protein levels in the cell lysates were measured. (E) The infectious titer in the culture media from HCV RNA-transfected cells.

To establish a Vero cell line that supported the entire HCV life cycle, miR-122, hSRBI, and hApoE were expressed in Vero cells via lentiviral transduction. After single-cell cloning, we obtained a Vero cell clone that expressed miR-122, hSRBI, and hApoE (Vero/miR122+SRBI+ApoE). The expression levels of these molecules in Vero/miR122+SRBI+ApoE cells were higher (miR122 and SRBI) or similar (ApoE) to the levels in Huh-7.5.1 cells (Fig. 5A and B). After HCVcc infection, HCV-positive cells were observed in Vero/miR122+SRBI+ApoE cells; however, the susceptibility of Vero/miR122+SRBI+ApoE cells to infection did not reach that of Huh-7.5.1 cells (Fig. 5C; see Fig. S3 in the supplemental material). After HCV RNA transfection, the intracellular HCV core Ag levels increased in a time-dependent manner in Vero/miR122+SRBI+ApoE cells (Fig. 5D), similar to that in Vero/miR122 cells (Fig. 1B). Furthermore, infectivity could be detected in the medium of Vero/miR122+SRBI+ApoE cells, although the infectivity titer in the medium of Vero/miR122+SRBI+ApoE cells was still lower than that in Huh-7.5.1 cells (Fig. 5E). These results indicated that the established Vero cell line expressing miR-122, SRBI, and ApoE together could support the entire life cycle of HCV.

Recently, SEC14L2 was reported as one of the host factors that enhance HCV replication in Huh-7.5 cells (24). Then, we tested the effect of SEC14L2 on HCV replication in Vero cells. The gene expression level of SEC14L2 in Vero/miR122+SRBI+ApoE cells was more than 10-fold higher than that in Huh-7.5.1 cells (see Fig. S4A in the supplemental material). Ectopic expression of human SEC14L2 increased intracellular HCV core Ag levels in Vero/miR122+SRBI+ApoE cells after HCV RNA transfection (Fig. S4B), suggesting that human SEC14L2 expression could enhance HCV replication in Vero cells.

## DISCUSSION

To establish a novel HCV cell culture system using Vero cells, we assessed several host factors for their contribution to each step of the HCV life cycle and identified three important molecules: miR-122, SRBI, and ApoE. The expression of miR-122 enhances the replication efficiency, SRBI is essential for infection, and ApoE is indispensable for the production of infectious HCV. The expression levels of these molecules in unmodified Vero cells were quite low compared to the levels in Huh-7.5.1 cells, which support efficient replication and the production of HCV.

We identified miR-122 as an essential factor for HCV replication in Vero cells. We obtained Vero/miR122 cells that highly expressed miR-122 via transduction with miR-122, and we observed HCV replication in these cells, although the level was still lower than that in Huh-7.5.1 cells. A possible explanation for this deficiency is the production of inhibitory factors for HCV replication in Vero cells. A point mutation that inactivates retinoic acid-inducible gene I (RIG-I) is present in Huh-7.5.1 cells and affects IFN signaling, rendering the cells unresponsive to IFN signal induction via RNA transfection. Vero cells are known to lack the gene cluster for type I IFN (25), and RNA transfection does not induce IFN signaling in these cells. However, in our experiments with Vero cells, type III IFN signal was induced by the transfection of HCV RNA (data not shown). Thus, the addition of a type III IFN antagonist might enhance HCV replication in these cells. It is also possible that other host factors associated with the efficient replication of HCV are still lacking in Vero cells, and supplementation with these substances might enhance HCV replication. For example, SEC14L2 is one of the host factors that were reported to enhance HCV replication in Huh-7.5 cells, and our data suggested that ectopic expression of human SEC14L2 could also enhance the HCV replication in Vero cells. Further studies are needed to identify such factors to obtain the maximum efficiency of HCV replication in Vero cells. In addition, the transfection efficiency of HCV RNA into Vero cells was quite low (data not shown), and this low transfection efficiency might be responsible for the lower replication efficiency of HCV.

We observed inconsistent results in infection studies with HCVpp and HCVcc. In Vero cells, the overexpression of CLDN1 enhanced HCVpp infection but not HCVcc infection. In contrast, the overexpression of SRBI did not enhance HCVpp infection but did enhance HCVcc infection. Likewise, in HEK293 cells, CLDN1 expression enhanced the efficiency of HCVpp infection to a level similar to that in Huh-7.5.1 cells, but there were not many infected cells after HCVcc infection, although HCV-positive cells were observed. These results suggest that the mechanism and other associated factors may be different for HCVpp and HCVcc, and the contribution of CLDN1 is more important in HCVpp infection. Such discrepancies between HCVpp and HCVcc have been previously reported (26, 27). HCVpp is a murine leukemia virus-based pseudotype virus that harbors the HCV E1 and E2 glycoproteins and is produced in nonhepatic cells that do not produce lipoproteins (16). Thus, the lipid composition on the surface of HCVpp is different from that of HCVcc, and the particle recognition and binding efficiency of SRBI, a high-density lipoprotein (HDL) receptor, might be affected. Therefore, we think that the HCVcc results more precisely represent the infection of HCV than the HCVpp results.

Based on our study of HCVcc infection, SRBI was identified as a necessary and sufficient factor for HCV infection in Vero cells. A functional analysis indicated that four HCV receptor molecules in Vero cells were equally functional when sufficiently expressed. Because SRBI expression was quite low, the additional expression of SRBI alone was sufficient to make Vero cells susceptible to HCV infection. Sourisseau et al. reported HCV infection in monkey hepatic cells derived from induced pluripotent stem cells from pigtail macaque (28). They reported that the HCV receptor molecules CD81, OCLN, CLDN1, and SRBI in pigtail macaques were functional in HCV infection. They also reported that CD81 derived from pigtail macaques showed lower susceptibility to infection of HCVpp and HCVcc than hCD81 did. The slightly lower susceptibility of vCD81 to infection compared to that of hCD81 was also observed in our Huh7-25-based assay for HCVpp infection. However, the overexpression of hCD81 in Vero cells did not cause susceptibility to HCVpp or HCVcc infection, indicating that CD81 is not the limiting factor for HCV infection in Vero cells.

The contribution of ApoE to the production of infectious HCV has been reported. A reduction in ApoE expression by small interfering RNAs remarkably suppressed the intra- and extracellular infectious virus production in an HCV cell culture system with HuH-7-derived cells (29). In our study, we found an essential role for ApoE in infectious virus production in Vero cells. The gene expression level of ApoE was low in Vero cells. A sequence analysis revealed that vApoE differs from the human orthologue by 18 amino acids. ApoE is a polymorphic protein with three major isoforms: ApoE2, ApoE3, and ApoE4. These three isoforms are distinguished by amino acid polymorphisms at position 130 (Cys or Arg [Cys/Arg]) and 176 (Cys/Arg). The polymorphisms of vApoE at position 130 (Arg) and 176 (Arg) are consistent with those of human ApoE4. Hishiki et al. reported that the ectopic expression of ApoE3 and ApoE4 efficiently supported infectious virus production, but the ectopic expression of ApoE2 did not (30). We compared the functions of hApoE and vApoE via transduction into HEK293 cells lacking ApoE expression. Overexpression of hApoE enabled the production of infectious virus, but overexpression of vApoE did not contribute to the production of infectious virus. Because ApoE contains many α-helix structures (31) and some of these α-helix regions were reported to be involved in the assembly of HCV particles (17, 32), these amino acid polymorphisms may affect the α-helix structures and infectious HCV particle formation. Although overexpression of hApoE enabled infectious virus production of HCV in Vero cells, the efficiency of Vero cells was still lower than that of Huh-7.5.1 cells. Because Vero cells are derived from monkey kidney, expression levels of other apolipoproteins in these cells are expected to be low. In addition to hApoE, the overexpression of some other apolipoproteins could enhance the infectious virus production of HCV in Vero cells.

HCV is a hepatotropic virus, and humans are the natural host of this virus. Because Vero cells are derived from monkey kidney, the factors identified in this study may pertain to host and organ tropisms. However, our data indicate that the investigated molecules in Vero cells, except for ApoE, were functional in susceptibility to HCV when sufficiently expressed. Furthermore, the sequence of mature miR-122 is conserved across species from fish to mammals (33). Therefore, in the case of Vero cells, the level of expression of these factors (miR-122, SRBI, and ApoE) is important for HCV susceptibility and restricts the host and organ tropisms. These data also indicate that these tropisms are regulated at each step of the virus life cycle: infection, replication, and infectious virus production. Although we identified the minimum essential factors for the completion of the HCV life cycle, the efficiency of each step did not reach the level attained in Huh-7.5.1 cells. Thus, the identification and supplementation of other enhancing factors for the HCV life cycle are necessary if the maximum efficiency of HCV propagation in Vero cells is to be attained; therefore, this nonhuman and nonhepatic cell line will also be useful for the elucidation of the mechanisms of HCV-host interactions.

Novel HCV cell culture systems using non-cancer-derived cells are necessary for the production of a vaccine against HCV because the current HCV cell culture system was generated from the hepatocellular carcinoma-derived HuH-7 cells. To establish a novel HCV cell culture system suitable for vaccine antigen production, we tested the replication capacity for HCV in several vaccine-producing cells, such as CHO cells, MDCK cells, MRC-5 cells, and Vero cells. However, none of these cells allowed HCV replication. Because it has been reported that several nonhepatic cell lines allowed HCV replication due to miR-122 expression (HEK293T cells [15, 16] and Hec1B cells [16]), we delivered miR-122 into Vero cells, which allowed HCV replication in Vero cells. Human kidney-derived HEK293 cells also supported HCV replication when miR-122 was expressed, but another human kidney-derived cell line, 786-O, did not support HCV replication even when miR-122 was expressed. Thus, miR-122 expression does not always lead to HCV replication in every cell line, and the existence of other factors involved in HCV replication is likely. Vero cells are recommended by the World Health Organization as cells for the production of vaccines for use in humans (34), and we successfully developed an HCV cell culture system using Vero cells that is useful for vaccine antigen production. However, some improvements will be necessary for practical use. For example, vaccine-producing Vero cells should be adapted to growth as a suspension in a serum-free medium for mass culture. Furthermore, higher virus production efficiency is required for purification.

In conclusion, we identified the minimum essential factors for the completion of the entire HCV life cycle and established a novel cell culture system with nonhuman nonhepatic Vero cells. This system will be useful not only for the further elucidation of HCV-host interaction mechanisms but also for HCV vaccine antigen production.

## MATERIALS AND METHODS

### Cells.

Vero C1008 cells (CRL-1586), HEK293 cells (CRL-1573), and 786-O cells (CRL1932) were purchased from the American Type Culture Collection (Manassas, VA). CHO cells (catalog no. 85050302) and MDCK cells (catalog no. 85011435) were purchased from the European Collection of Authenticated Cell Cultures (Salisbury, United Kingdom). MRC-5 cells (catalog no. 02-021) were purchased from DS Pharma Biomedical (Osaka, Japan). The cured cell line, Huh-7.5.1 cells, were a kind gift from Francis V. Chisari (Scripps Research Institute, La Jolla, CA) (12).

### Sequencing and cloning of host factors.

Sequencing and cloning of host factors are described in Text S1 in the supplemental material.

### Expression vectors.

The construction of expression vectors is described in Text S1 in the supplemental material.

### Lentiviral transduction.

Lentivirus was generated with pLVSIN vectors and pSEC14L2-BlastR (24), a kind gift from Charles M. Rice (The Rockefeller University, New York, NY), and the Lenti-X HTX packaging system (Clontech, Mountain View, CA). Lentiviral transduction was performed according to the manufacturer’s instructions.

### HCV RNA synthesis and RNA transfection.

The plasmid encoding a chimeric full-length genomic HCV RNA, pJ6/JFH-1, was obtained from pJFH1 (GenBank accession number AB047639) (10) by replacement of the core-to-p7 region (AgeI-BclI) of pJ6CF (GenBank accession number AF177036), a kind gift from Jens Bukh (Copenhagen University Hospital, Hvidovre, Denmark). RNA synthesis and transfection were performed as previously described (35, 36).

### Virus stock.

HCVcc used in this study was the cell culture-adapted JFH-1 virus (27).

### Quantification of HCV core protein.

The concentration of the HCV core protein in the culture medium and the cell lysate was measured using a chemiluminescent-enzyme immunoassay (Lumipulse Ortho HCV antigen; Fujirebio, Tokyo, Japan) in accordance with the manufacturer’s instructions (37).

### Gene expression analysis.

The assessment of expression levels of host factors was performed as described previously. The gene-specific primer and probe sets we used for the detection of CD81, OCLN, CLDN1, SRBI, and ApoE detect both human and monkey orthologues. We also used another gene-specific primer and probe set for human ApoE that detected only human ApoE. The miRNA expression level was measured as previously described (38).

### Single-cell cloning by limiting dilution.

Lentivirus-transduced Vero cells were diluted with medium at 2.5 cells/ml and seeded at 200 µl/well in 96-well plates. The subclones obtained were expanded and stored at −80°C pending further characterization.

### Determination of the infectivity titer.

Determination of the infectivity of the culture supernatants is described in Text S1 in the supplemental material.

### Immunostaining.

The methods of immunostaining are described in Text S1 in the supplemental material.

### Virus entry assay.

The HCV pseudotype virus (HCVpp) harboring the JFH-1 E1 and E2 glycoproteins was prepared as previously described (39). A virus entry assay using HCVpp was performed as previously described (38).

### Immunoblotting.

The methods of immunoblotting are described in Text S1 in the supplemental material.

### Statistical analysis.

Significant differences were evaluated using Student’s *t* test. *P* values of <0.05 were considered significant.

## SUPPLEMENTAL MATERIAL

Text S1 Supplemental Materials and Methods. Supplemental Materials and Methods and references are given. Download Text S1, DOCX file, 0.05 MB

Figure S1  Functional characterizations of HCV receptor molecules originally expressed in Vero cells and Huh-7.5.1 cells. (A) Relative gene expression levels of the HCV receptors in Huh7-25 cells, 786-O cells, HEK293 cells, and Huh-7.5.1 cells. The results are expressed as the fold difference in expression compared to the level in Huh-7.5.1 cells. The dashed lines indicate the mRNA expression level in Huh-7.5.1 cells. (B) Comparisons of the miR-122 expression levels in Huh-7.5.1 cells, Huh7-25 cells, 786-O cells, 786-O/miR122 cells, HEK293 cells, and HEK293/miR122 cells. The results are expressed as the fold difference in expression compared to the level in Huh-7.5.1 cells. The dashed lines indicate the miR-122 expression level in Huh-7.5.1 cells. (C and D) HCV replication in 786-O/miR122 cells (C) and in HEK293/miR122 cells (D). HCV RNA was electroporated into these cells, and the HCV core protein levels in the cell lysates were measured. Independent assays were performed in triplicate, and the results are presented as the means ± standard deviations (error bars). Download Figure S1, TIF file, 0.2 MB

Figure S2  HCV infection of cells expressing HCV receptors derived from human and Vero cells. (A) HCVcc infection of Huh7-25 cells expressing hCD81 or vCD81, HEK293 cells expressing hCLDN1 or vCLDN1, and Vero cells expressing hSRBI or vSRBI. HCV-infected foci were counted and expressed as focus-forming units (FFU) per milliliter. (B) HCVcc infection of Vero cells expressing hCD81, hOCLN, hCLDN1, and hSRBI. HCV-infected foci were counted and expressed as FFU per milliliter. Abbreviations: nd, not detected; ns, not significant. Download Figure S2, TIF file, 0.2 MB

Figure S3  HCVcc infection of Vero cells, Vero/miR122+SRBI+ApoE cells, and Huh-7.5.1 cells. HCV-infected foci were counted and expressed as focus-forming units (FFU) per milliliter. nd, not detected; ns, not significant. Download Figure S3, TIF file, 0.1 MB

Figure S4  Effect of expression of SEC14L2 on HCV replication in Vero cells. (A) Expression levels of SEC14L2 in Vero/miR122+SRBI+ApoE cells, Vero/miR122+SRBI+ApoE+LV-SEC14L2 cells, and Huh-7.5.1 cells. (B) HCV RNA was electroporated into the cells, and the HCV core protein levels in cell lysates were measured. Download Figure S4, TIF file, 0.1 MB
