# Supplementary material for: Completion of the Entire Hepatitis C Virus Life Cycle in Vero Cells Derived from Monkey Kidney
Source: mBio. 2016 Jun 14;7(3):e00273-16. doi: 10.1128/mBio.00273-16 (PMC4916372; doi:10.1128/mBio.00273-16)
Supplement: Text S1 — Supplemental Materials and Methods. Supplemental Materials and Methods and references are given. Download [file mbo003162864s1.docx]

**Supplementary Information**

**Completion of the Entire Hepatitis C Virus Life Cycle**

**in Vero Cells Derived from Monkey Kidney**

Asako Murayama, Nao Sugiyama, Takaji Wakita, Takanobu Kato.

**SUPPLEMENTARY MATERIALS AND METHODS**

**Sequencing and cloning of host factors.** For the sequencing of host factors expressed in Vero cells or in Huh-7.5.1 cells, total cellular RNA was extracted from the cells using the RNeasy Mini RNA kit (QIAGEN, Hilden, Germany). cDNA was synthesized from total cellular RNA using SuperScript III Reverse Transcriptase (Invitrogen, Carlsbad, CA) and a random primer (TaKaRa Bio, Shiga, Japan). The open reading frames and a part of the UTRs of each host factor were amplified by nested PCR using TaKaRa LA Taq (TaKaRa Bio) and appropriate primers in the 5’UTR and the 3’UTR of each host factor. The sequence was determined directly from a PCR-amplified fragment using internal primers.

**Expression vectors.** To generate the miR-122 expression plasmid pLVSIN-miR122, the β-globin intron region including the human miR-122 precursor sequence was amplified from the miRNASelect pEGP-mmu-mir-122 Expression Vector (Cell Biolabs, San Diego, CA) using sense and antisense primers containing *Xba*I and *Not*I sites, respectively, and cloned into the pLVSIN-EF1α Neo (TaKaRa Bio) vector in the *Xba*I and *Not*I sites. To generate the host factor expression plasmids, the ORF fragment of each host factor except for human CD81 and human Apolipoprotein E (ApoE) were amplified from cDNA synthesized from the total cellular RNA of Vero cells or Huh-7.5.1 cells. The ORF of human CD81 was amplified from the pcDNA3.1-CD81 plasmid ([1](#_ENREF_1)), a kind gift from Dr. Yoshiharu Matsuura (Osaka University, Osaka, Japan). The ORF of human ApoE was amplified from the ApoE3 plasmid ([2](#_ENREF_2)), a kind gift from Dr. Kunitada Shimotohno (National Center for Global Health and Medicine, Chiba, Japan). The sense primers for CD81, Occludin (OCLN), Claudin-1 (CLDN1), Scavenger receptor class B type I (SRBI) and ApoE contain an *Xba*I site, the antisense primers for CD81, OCLN, CLDN1, and SRBI contain a *Not*I site and the antisense primer for ApoE contains a *Bam*HI site for cloning purposes. PCR fragments were cleaved with the appropriate restriction enzymes and cloned into the pLVSIN-EF1α Neo (TaKaRa Bio) vector. SEC14L2 expression vector, pSEC14L2-BlastR ([3](#_ENREF_3)), was provided by Dr. Charles M. Rice (The Rockefeller University, New York, NY). The sequences were confirmed by nucleotide sequencing.

**Determination of the infectivity titer.** Determination of the infectivity of the culture supernatants of HCV RNA-transfected cells was performed as described previously ([4](#_ENREF_4)) with a slight modification. When necessary, the culture medium was concentrated 30-fold using an Amicon Ultra-15 spin column (100 kDa cut-off, Millipore, Bedford, MA). The HCV-infected foci were visualized by staining with anti-NS5A antibody, clone KS0265-1 ([4](#_ENREF_4)), and Alexa Fluor 488 Goat Anti-mouse IgG (Invitrogen). The infectivity titer was quantified by counting the stained foci and expressed the value as the number of focus-forming units per milliliter (FFU/mL) or per dish (FFU/dish).

**Immunostaining.** The target cells were seeded at 1 × 10^5^ cells/well on glass cover slips in a 12-well plate and infected with HCVcc. Three days after infection, the cells were fixed in 4% paraformaldehyde and permeabilized. After blocking, the HCV-positive cells were visualized by staining with anti-NS5A antibody and Alexa Fluor 488 Goat Anti-mouse IgG, and the nuclei were stained with 4',6-diamidino-2-phenylindole (DAPI).

**Immunoblotting.** Ten micrograms of each lysate was loaded onto SDS-PAGE gels and analyzed by immunoblots. The antibodies that we used for the immunoblots were mouse monoclonal anti-Human CLA-1 (SR-BI, 610883; BD Biosciences, San Jose, CA), goat polyclonal anti-Apolipoprotein E Antibody (AB947; Millipore) and mouse anti-β-actin (A5441; Sigma-Aldrich, St. Louis, MO). The signal was detected using the HRP-linked Anti-mouse IgG (Cell signaling, Danvers, MA) and the HRP-linked anti-goat IgG (Sigma-Aldrich, St. Louis, MO), with enhanced chemiluminescence reagents (SuperSignal West Pico Chemiluminescent Substrate; Thermo Fisher Scientifc, Waltham, MA).

**SUPPLEMENTARY REFERENCES**

1. **Akazawa D, Date T, Morikawa K, Murayama A, Miyamoto M, Kaga M, Barth H, Baumert TF, Dubuisson J, Wakita T.** 2007. CD81 expression is important for the permissiveness of Huh7 cell clones for heterogeneous hepatitis C virus infection. J Virol **81:**5036-5045.

2. **Hishiki T, Shimizu Y, Tobita R, Sugiyama K, Ogawa K, Funami K, Ohsaki Y, Fujimoto T, Takaku H, Wakita T, Baumert TF, Miyanari Y, Shimotohno K.** 2010. Infectivity of hepatitis C virus is influenced by association with apolipoprotein E isoforms. J Virol **84:**12048-12057.

3. **Saeed M, Andreo U, Chung HY, Espiritu C, Branch AD, Silva JM, Rice CM.** 2015. SEC14L2 enables pan-genotype HCV replication in cell culture. Nature **524:**471-475.

4. **Murayama A, Sugiyama N, Yoshimura S, Ishihara-Sugano M, Masaki T, Kim S, Wakita T, Mishiro S, Kato T.** 2012. A subclone of HuH-7 with enhanced intracellular hepatitis C virus production and evasion of virus related-cell cycle arrest. PLoS One **7:**e52697.
