# Supplementary figures and images for: Completion of the Entire Hepatitis C Virus Life Cycle in Vero Cells Derived from Monkey Kidney
Source: mBio. 2016 Jun 14;7(3):e00273-16. doi: 10.1128/mBio.00273-16 (PMC4916372; doi:10.1128/mBio.00273-16)

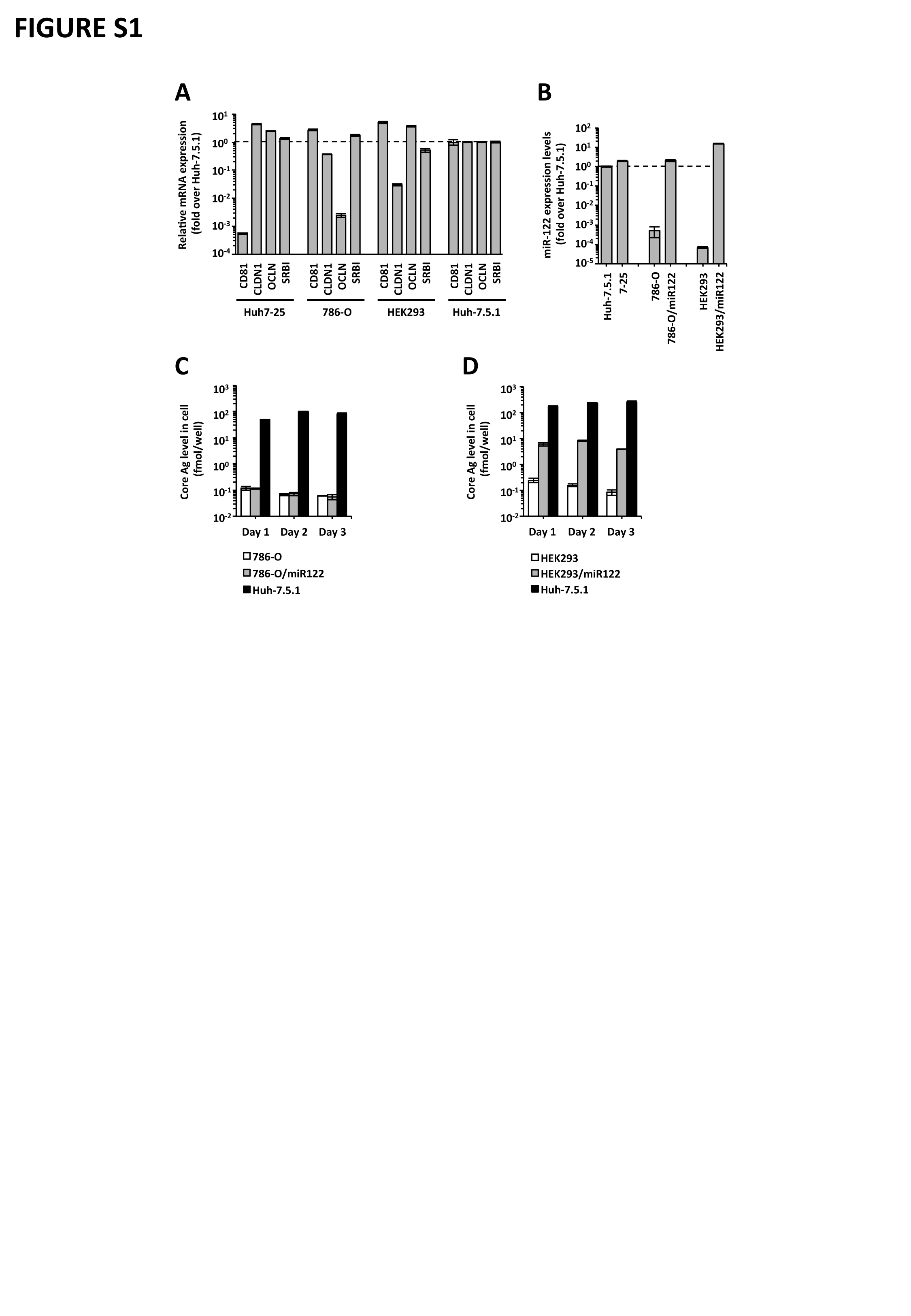

Supplement: Figure S1 — Functional characterizations of HCV receptor molecules originally expressed in Vero cells and Huh-7.5.1 cells. (A) Relative gene expression levels of the HCV receptors in Huh7-25 cells, 786-O cells, HEK293 cells, and Huh-7.5.1 cells. The results are expressed as the fold difference in expression compared to the level in Huh-7.5.1 cells. The dashed lines indicate the mRNA expression level in Huh-7.5.1 cells. (B) Comparisons of the miR-122 expression levels in Huh-7.5.1 cells, Huh7-25 cells, 786-O cells, 786-O/miR122 cells, HEK293 cells, and HEK293/miR122 cells. The results are expressed as the fold difference in expression compared to the level in Huh-7.5.1 cells. The dashed lines indicate the miR-122 expression level in Huh-7.5.1 cells. (C and D) HCV replication in 786-O/miR122 cells (C) and in HEK293/miR122 cells (D). HCV RNA was electroporated into these cells, and the HCV core protein levels in the cell lysates were measured. Independent assays were performed in triplicate, and the results are presented as the means ± standard deviations (error bars). Download [file mbo003162864sf1.tif]

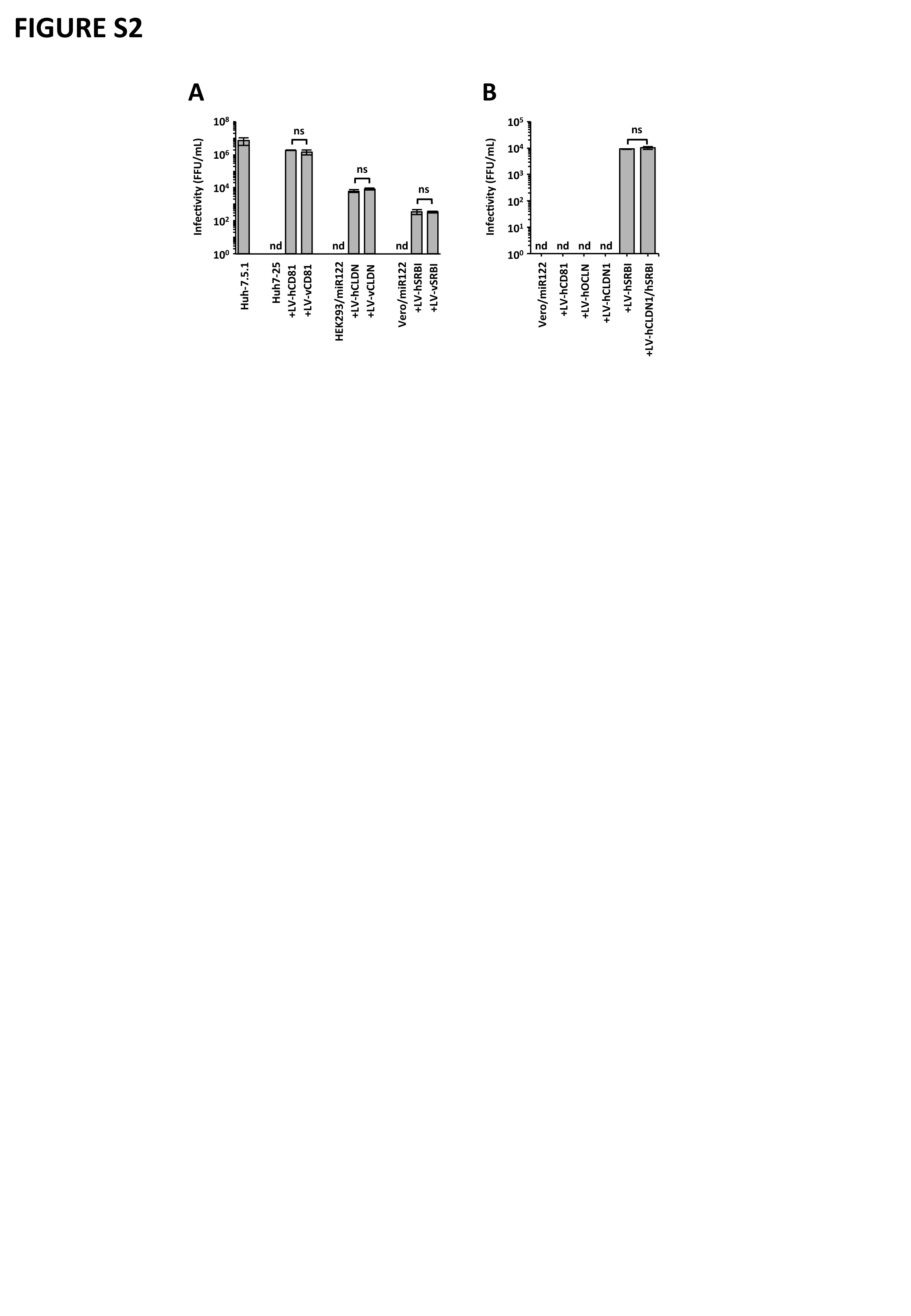

Supplement: Figure S2 — HCV infection of cells expressing HCV receptors derived from human and Vero cells. (A) HCVcc infection of Huh7-25 cells expressing hCD81 or vCD81, HEK293 cells expressing hCLDN1 or vCLDN1, and Vero cells expressing hSRBI or vSRBI. HCV-infected foci were counted and expressed as focus-forming units (FFU) per milliliter. (B) HCVcc infection of Vero cells expressing hCD81, hOCLN, hCLDN1, and hSRBI. HCV-infected foci were counted and expressed as FFU per milliliter. Abbreviations: nd, not detected; ns, not significant. Download [file mbo003162864sf2.tif]

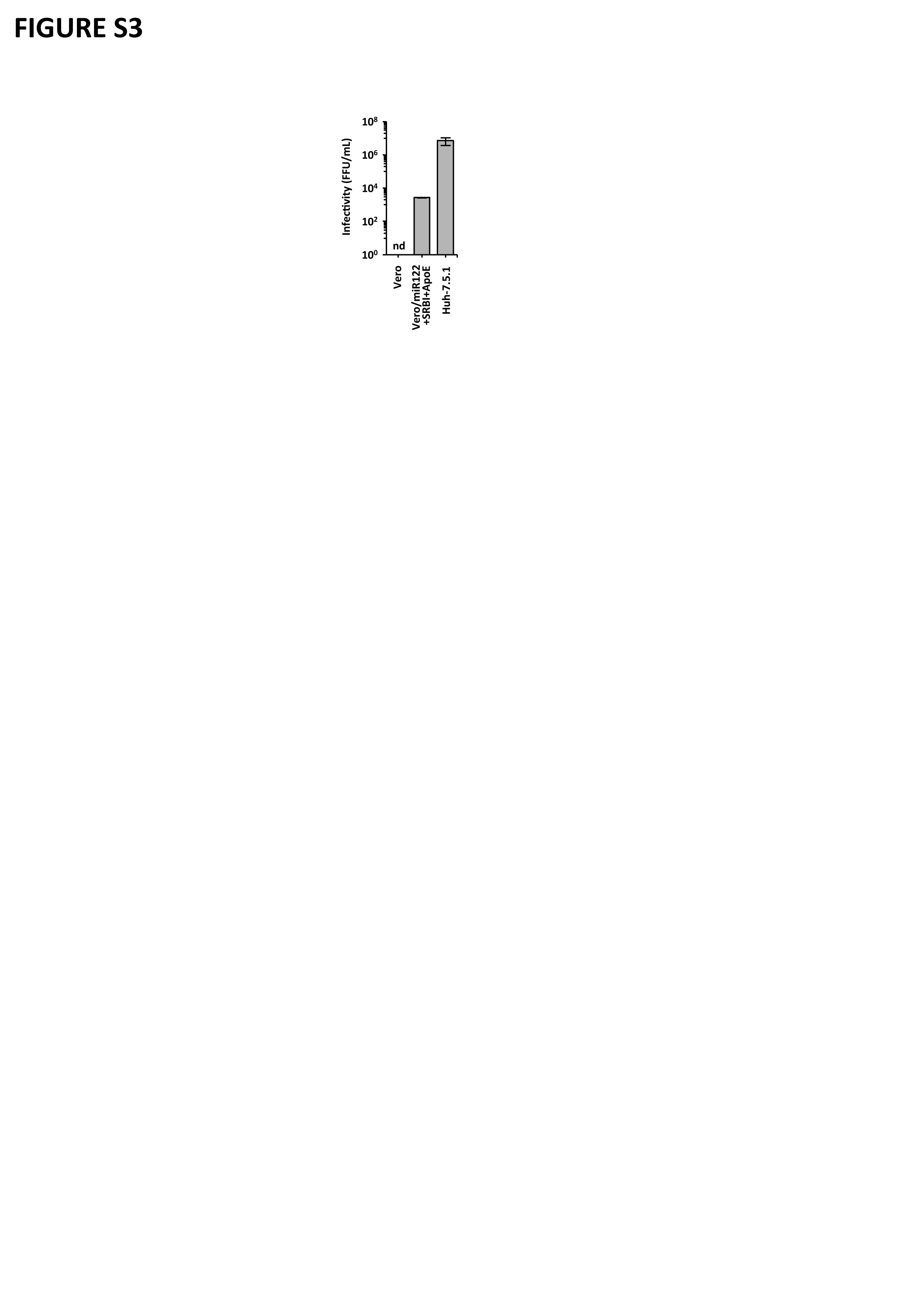

Supplement: Figure S3 — HCVcc infection of Vero cells, Vero/miR122+SRBI+ApoE cells, and Huh-7.5.1 cells. HCV-infected foci were counted and expressed as focus-forming units (FFU) per milliliter. nd, not detected; ns, not significant. Download [file mbo003162864sf3.tif]

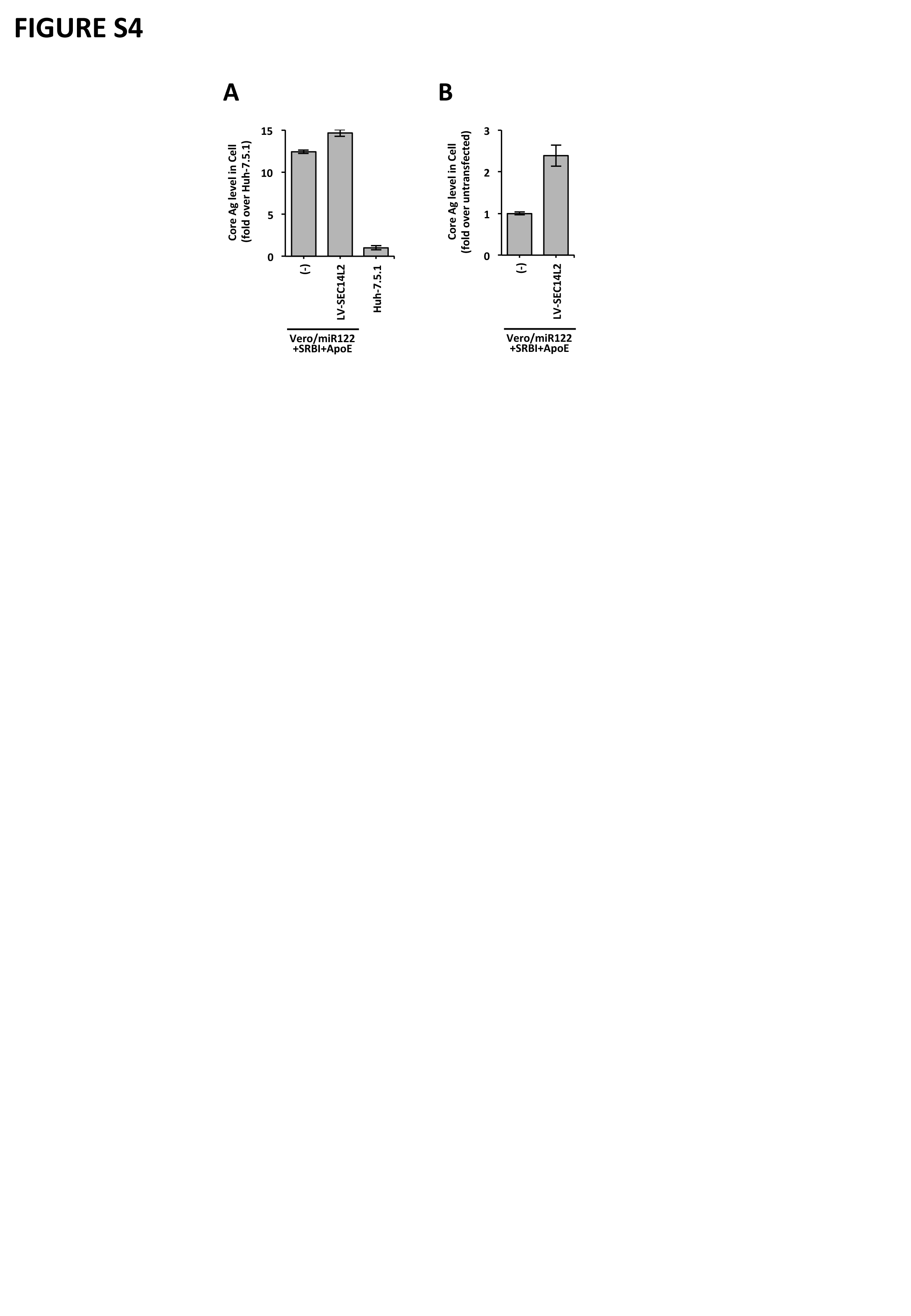

Supplement: Figure S4 — Effect of expression of SEC14L2 on HCV replication in Vero cells. (A) Expression levels of SEC14L2 in Vero/miR122+SRBI+ApoE cells, Vero/miR122+SRBI+ApoE+LV-SEC14L2 cells, and Huh-7.5.1 cells. (B) HCV RNA was electroporated into the cells, and the HCV core protein levels in cell lysates were measured. Download [file mbo003162864sf4.tif]
